# Radiation-Induced Skin Injuries to Patients: What the Interventional Radiologist Needs to Know

**DOI:** 10.1007/s00270-017-1674-5

**Published:** 2017-05-11

**Authors:** Werner Jaschke, Matthias Schmuth, Annalisa Trianni, Gabriel Bartal

**Affiliations:** 1 Department of Radiology, Medical University Innsbruck, Anichstrasse 35, 6020 Innsbruck, Austria; 2 Department of Dermatology, Venereology and Allergology, Medical University Innsbruck, Anichstrasse 35, 6020 Innsbruck, Austria; 3 Department of Physics, Udine University Hospital, Piazzale S. Maria Della Misericordia, n. 15, 33100 Udine, Italy; 4 Department of Radiology, Meir Medical Center, Street Tchernichovsky 59, 44281 Kfar Saba, Israel

**Keywords:** Interventional radiology, Radiation, Skin injuries

## Abstract

For a long time, radiation-induced skin injuries were only encountered in patients undergoing radiation therapy. In diagnostic radiology, radiation exposures of patients causing skin injuries were extremely rare. The introduction of fast multislice CT scanners and fluoroscopically guided interventions (FGI) changed the situation. Both methods carry the risk of excessive high doses to the skin of patients resulting in skin injuries. In the early nineties, several reports of epilation and skin injuries following CT brain perfusion studies were published. During the same time, several papers reported skin injuries following FGI, especially after percutaneous coronary interventions and neuroembolisations. Thus, CT and FGI are of major concern regarding radiation safety since both methods can apply doses to patients exceeding 5 Gy (National Council on Radiation Protection and Measurements threshold for substantial radiation dose level). This paper reviews the problem of skin injuries observed after FGI. Also, some practical advices are given how to effectively avoid skin injuries. In addition, guidelines are discussed how to deal with patients who were exposed to a potentially dangerous radiation skin dose during medically justified interventional procedures.

## Introduction

Radiation injuries were primarily observed in the pioneering days of radiology when the biological effects of radiation were not yet understood and radiation protection was unavailable. The first case of human radiogenic dermatitis of the hand was reported in January 1896 [1]. In 1925, several patients suffering from radiation-induced skin injuries were reported by Groedel [2]. By taking preventive measures, radiation injuries due to medical imaging were completely eliminated within 30 years after the introduction of procedures utilizing X-rays into medicine [1]. Exposures of patients exceeding 100 mSv effective dose were extremely rare in medical imaging until the introduction of multislice CT and fluoroscopically guided interventions (FGI). Thus, CT and FGI are of major concern regarding radiation safety in medical imaging [3–6]. CT and fluoroscopy account for approximately 10% of all imaging procedures, but contribute approximately 80% to the mean collective dose. The number of fluoroscopically guided interventions increased dramatically during the last 30 years and continues to rise [4, 7]. In some countries, numbers doubled every 2–4 years [1, 8, 9]. For example, percutaneous coronary interventions (PCI) are performed with a frequency of approx. 4500/1 Mill. inhabitants in Germany (http://www.gbe-bund.de/PCI). Furthermore, FGI of the lower extremities is another growing field. The prevalence of peripheral arterial occlusive disease (PAOD) is estimated at 3–10% in the general population; a percentage that is even higher among the population aged 70 and older (15–20%) [10]. The incidence in less developed and developed countries increased within the last 10 years by a rate of 28,7% and 13,1%, respectively [10, 11]. Most of these patients will undergo a percutaneous procedure at some stage of their disease. It is, therefore, not surprising that the number of endovascular procedures is continuously increasing. The first radiation-induced skin injuries associated with PCI were reported in the early nineties [8, 12–14]. Radiation-induced skin injuries and epilation were the most commonly reported side effects following procedures with uncommonly high radiation exposure, mostly resulting from CT perfusion studies of the brain and percutaneous coronary interventions (PCI). In addition, there is an increasing concern about the biological long-term effects of low-level radiation affecting staff and patients [15–18]. Thus, more than a hundred years after the discovery of X-rays, the subject of radiation protection has again emerged as a major concern of the public, medical professionals and health authorities [3].

This paper reviews the most important tissue injuries observed after FGI. The main contribution of this paper is the observation and analysis of skin injuries, as radiation-induced cataractogenesis was just recently covered by a review article in this journal [19]. Moreover, some practical advice is given regarding the effective avoidance of skin injuries.

## Radiation-Induced Tissue Injuries

Radiation-induced tissue injuries were previously labeled deterministic effects of radiation. The most important tissue injuries affect the skin and the eye lens. Typically, radiation-induced skin injuries occur after a time delay of days, sometimes weeks following a procedure, in which a threshold of skin exposure has been exceeded (Table 1) [8, 20, 21].

**Table 1:** Radiation-induced lesions of the skin and eye lens with respect to dose and time of onset. Adapted from ICRP publication 85/2000 [8]

Effect	Approximate threshold dose (Gy)	Time of onset
Skin		
Early transient erythema	2	2–24 h
Main erythema reaction	6	~1.5 weeks
Temporary epilation	3	~3 weeks
Permanent epilation	7	~3 weeks
Dry desquamation	14	~4 weeks
Moist desquamation	18	~4 weeks
Secondary ulceration	24	>6 weeks
Late erythema	15	8–10 weeks
Ischemic dermal necrosis	18	>10 weeks
Dermal atrophy (1st phase)	10	>52 weeks
Telangiectasis	10	>52 weeks
Dermal necrosis (delayed)	>12	>52 weeks
Skin cancer	Unknown	>15 years

The potential risk of the general patient population for exposure to a radiation dose above a substantial level of 3 Gy skin dose (Table 2) has increased over the years [22].

**Table 2:** Substantial radiation dose levels which should trigger follow-up of patients in order to detect clinically relevant skin reactions. Adapted from NCRP report Nr 168 (2010)

Peak skin dose	3 Gy
Cumulative air KERMA at RP	5 Gy
Kerma area product	500 Gy cm^2^
Fluoroscopy time	60 min^a^

*NCRP* National Council on Radiation Protection and Measurements, Bethesda, USA

^a^ Institutions performing procedures with potentially high dose levels shall measure and record dose metrics, and shall not rely on fluoroscopy time alone

The reasoning behind this approach is that FGI procedures are more often used, more complex, more frequent and longer lasting. Moreover, the patients are more frequently obese, and obesity is a significant contributing factor to higher exposure. In addition, patients undergoing several interventional procedures in their lifetime are more frequently encountered.

**Fig. 1:**
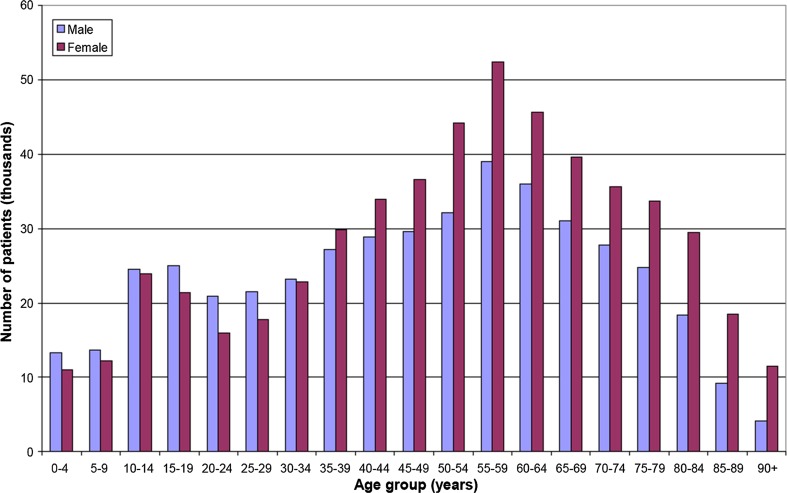
Number of patients in Denmark having one or more X-ray examinations in 2004 as a function of age and sex (adapted from [18])

**Fig. 2:**
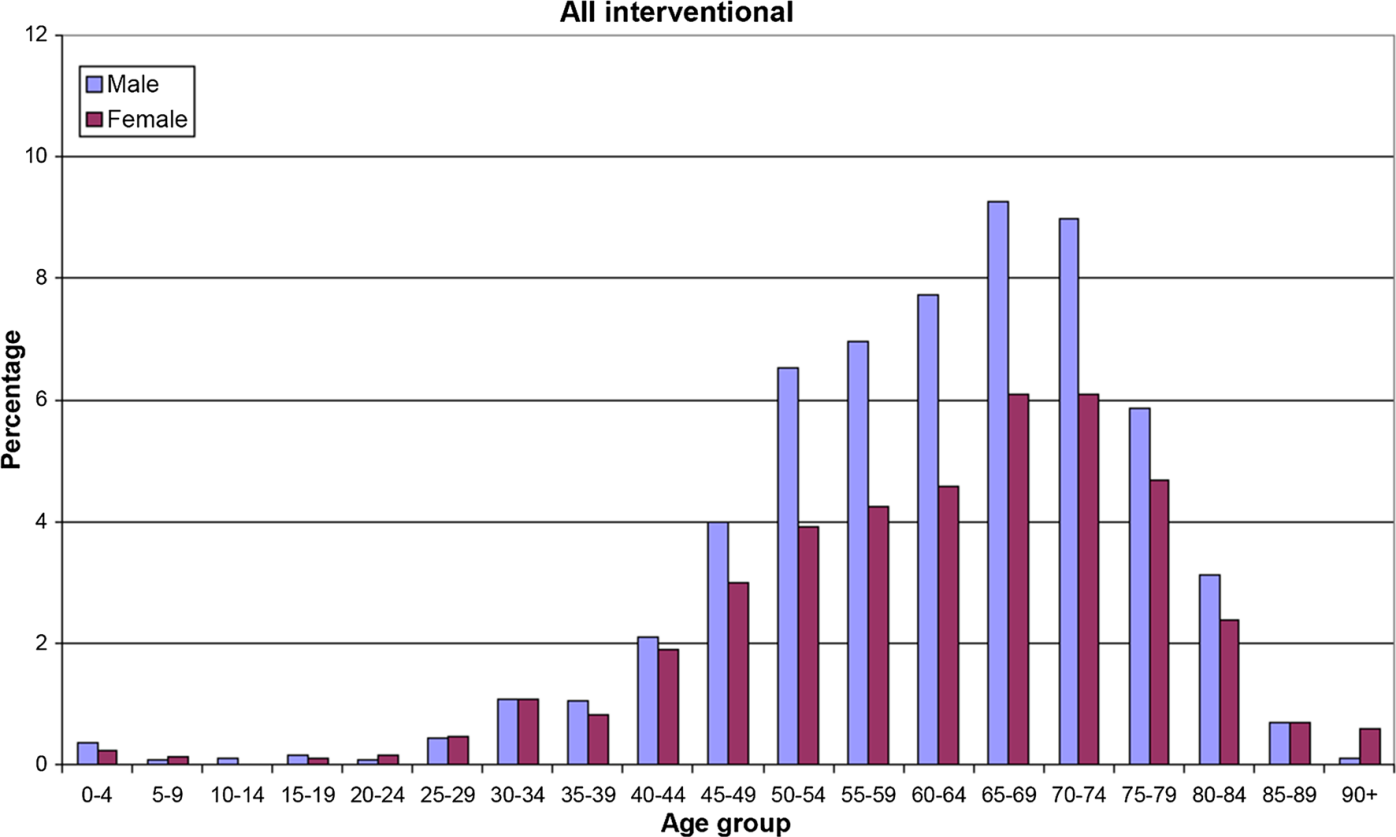
Age distribution of patients in Denmark undergoing FGI procedures

The heavy bias toward elderly patients having X-ray examinations and interventional procedures is shown in Figs. 1 and 2 [23]. Patients at risk for tissue injuries are typically of older age (55–85 years) and suffering from chronic diseases—consequentially requiring multiple imaging and interventional procedures.

## Radiation-Induced Skin Injuries

Severe skin injuries from fluoroscopically guided procedures are either still rare, or underreported at present. In 1994, the US Food and Drug Administration (FDA) received approximately 40 separate reports [24]. Radiation-induced ulcers are currently reported in less than 1% of all patients undergoing cardiac FGI [25]. Skin reactions related to radiation exposure can be distinguished as either prompt/acute/subacute (from 24 h up to 2 months) or chronic (more than 2 months up to years) [1, 21, 26]. Prompt radiation-induced skin reactions occur within less than 2 weeks. The most common prompt skin reaction is an erythematous reaction which can occur from a few hours up to 24 h after exposure of more than 2 Gy. This complication is rarely reported in specialist literature, but actually quite commonly observed after long and complex interventional procedures. Acute radiation injury of the skin is characterized by erythema with vesicles, erosion, temporary epilation and pain and itching persisting up to 9 weeks. Chronic radiation injury of the skin (CRIS) presents with an insidious and variable onset of symptoms ranging from erythema, atrophy, epilation, telangiectasia and pruritus, as well as pain due to dermal necrosis and ulceration. CRIS occurs typically months to years after several high-dose radiation exposures or a single very high radiation exposure with a cumulative peak skin dose threshold of 10 Gy. Clinically, the typical patient with CRIS presents with permanent erythema, dermal atrophy and ulceration. An overview of skin lesions, time of onset, development over time and relation to peak skin dose is given in Table 3.

**Table 3:** Cutaneous radiation injury: grading, threshold dose and timing

Grade	Skin dose^a^	Prodromal stage	Latent stage	Manifest illness stage	Third wave of erythema^b^	Recovery	Late effects
I	>2 Gy (200 rad)^c^	1–2 days post-exposure or not seen	No injury evident for 2–5 weeks post-exposure^d^	2–5 weeks post-exposure, lasting 20–30 days: redness of skin, slight edema, possible increased pigmentation 6–7 weeks post-exposure, dry desquamation	Not seen	Complete healing expected 28–40 days after dry desquamation (3–6 months post-exposure)	Possible slight skin atrophy Possible skin cancer exposure
II	>15 Gy (1500 rad)	6–24 h post-exposure with immediate sensation of heat lasting 1–2 days	No injury evident for 1–3 weeks post-exposure	1–3 weeks post-exposure; redness of skin, sense of heat, edema, skin may turn brown 5–6 weeks post-exposure, edema of subcutaneous tissues and blisters with moist desquamation possible epithelialization later	10–16 weeks post-exposure, injury of blood, vessels, edema and increasing pain Epilation may subside, but new ulcers and necrotic changes are possible	Healing depends on size of injury and the possibility of more cycles of erythema	Possible skin atrophy or ulcer recurrence Possible telangiectasia (up to 10 years post-exposure) Possible skin cancer decades after exposure

Dose range is given for patients with normal radiosensitivities in the absence of mitigating or aggravating physical or clinical factors. Response to radiation does not apply to the skin of the scalp. Threshold dose and timing are not absolute values, but rather the best appraisal values. Signs and symptoms are expected to appear earlier as skin dose increases

Taken from: Cutaneous radiation injury: factsheet for physicians. CDC Stacks/Center of Disease Control and Prevention, USA; https://stacks.cdc.gov/view/cdc/23969 [26]

^a^ Absorbed dose to at least 10 cm^2^ of the basal cell layer of the skin

^b^ Especially with beta exposure

^c^ The Gray (Gy) is a unit of absorbed dose and reflects an amount of energy deposited in a mass of tissue (1 Gy = 100 rad)

^d^ Skin of the face, chest and neck will have a shorter latent phase than the skin of the palms of the hands and the skin of the feet

It is important to note that CRIS is not always preceded by an acute skin injury or that a previous minor skin reaction was not detected during initial treatment sessions. A skin lesion may, therefore, not be attributed to a previous radiation exposure. In addition, patients and physicians are often unaware of radiation-induced complications of interventional procedures. Some patients may even be unaware that endovascular procedures are performed under fluoroscopic guidance.

Radiation-induced skin ulcer is the most severe form of radiation-induced dermatitis. The incidence of radiation-induced ulcers is not as rare as previously assumed [25]. Radiation-induced skin ulcer is a consequence of an excessively high cumulative skin dose. The correct diagnosis is difficult because the ulcers occur with a considerable time delay of months or even years after exposure, and the causation is not always obvious. Usually, patients do not directly consult the interventionalist, but rather a primary care physician or a dermatologist who may be unaware of previous radiation exposures. Ulcers can be triggered by minor trauma caused by scratching, applying topical agents or hot packing to relieve radiation-induced pruritus or pain.

Obesity, diabetes, nicotine abuse, previous radiation exposure in the same body region, compromised skin integrity, Fitzpatrick skin type I–II (fair skin), diabetes, autoimmune/connective tissue disease(for example scleroderma, lupus erythematosus and mixed connective tissue disease), hyperthyroidism and certain drugs are among many other factors predisposing to an increased radiosensitivity at lower radiation doses [14, 27, 21, 28–30]. The relative contribution of nutritional status or preoperative skin integrity is under debate [31].

Malfunctioning of DNA repair genes (*ataxia* teleangiectasia, xeroderma pigmentosum) and chemotherapy are additional risk factors for radiation-induced skin injuries [28, 29, 32, 33]. Patients suffering from ataxia teleangiectasia carry an autosomal recessive ATM gene. It has been suggested that heterozygous gene carriers (approx. 1% of population) carry a higher risk for radiation-induced skin injuries [21]. Genetic disorders which are connected to higher radiosensivity are listed in Table 4.

**Table 4:** Genetic disorders increasing radiosensitivity [21, 34]

Ataxia teleangiectatica
ATM-like disorder
Nijmegen breakage syndrome
Severe combined immune deficiency (SCID)
Ligase IV syndrome
Seckel syndrome
Fanconi anemia
Bloom syndrome
Gorlin syndrome
Familiar polyposis
Gardner syndrome
Hereditary melanoma
Dysplastic nervus syndrome
Xeroderma pigmentosum variant

A number of drugs increase radiosensitivity. The most important drugs which are known to increase radiosensitivity are listed in Table 5.

**Table 5:** Drugs increasing radiosensitivity [14, 20–22, 34, 35]

Actinomycin D
Doxorubicin
Bleomycin
5-FU
Methotrexat
NNRTI-based antiretroviral therapy in HIV patients
Platinum containing chemotherapeutic drugs
Antiangiogenic drugs
BRAF inhibitors and others

Radiation recall refers to inflammation and other reactions developing in previously irradiated areas that are subsequently exposed to a second agent. Radiation recall reactions have been attributed to a wide range of cytotoxic drugs since they were first reported with actinomycin D. These include taxanes, anthracyclines, cytarabine, bleomycin, capecitabine, vinblastine, etoposide, methotrexate, melphalan, dacarbazine, oxaliplatin, hydroxyurea, 5-fluorouracil and IFN. Other noncytotoxic agents such as simvastatin, isoniazid, rifampicin, pyrazinamide and tamoxifen have also been under suspicion. Re-irradiation of a previously irradiated area may also cause a similar response.

## Pathophysiology of Radiation-Induced Skin Reactions and Injuries

On histologic examination, morphological findings depend on the phase of radiation injury. The immediate and delayed erythema is accompanied by widening of the rete ridges, edematous swelling of the dermis, dilatation of the dermal vessels, swelling of the endothelia and fibrous thickening of the vessel walls, first precipitating erythema and then teleangiectases [36–38]. Intravascular thromboses and erythrocyte extravasation have also been described. Atrophy of the epidermis and adnexal structures (hair follicles, sebaceous glands and sweat glands), and/or degeneration of basal keratinocytes are found at later stages and correlate with hair loss [39]. In addition, dermal collagen fibers appear coarse and increased in number. Hyperpigmentary changes correlate with an increase in dermal melanophages [40].

On the molecular level, depending on the absorbed energy, ionizing radiation can break chemical bonds and cause ionization of molecules such as DNA, membrane lipids, proteins and even water [41]. Because ionizing irradiation affects the cell cycle, DNA damage occurs primarily in the proliferating epidermal keratinocytes of the basal cell layer, resulting in various types of cell death (apoptosis, necrosis) [42, 43]. This process is accompanied by the secretion of the second messengers including inflammatory mediators (e.g., cytokines, chemokines and prostaglandins). In the dermis, these inflammatory mediators cause changes in vessel endothelia, fibroblast proliferation and collagen production. The final result of exposure to ionizing radiation is skin inflammation [44, 45]. In severe cases of radiation exposure, toxins and/or unrestricted inflammation can result in overt destruction of the epidermis [46]. Following restoration of epidermal integrity, long-term effects of skin irradiation comprise increased risk of skin cancers, hyperkeratoses, cutaneous atrophy, hair loss (epilation), telangiectasia, hemangiomas and fibrosis [47–50].

In mild to moderate cases, cytokine release during tissue inflammation indirectly results in impairment of the epidermal permeability barrier [51]. Damage to the permeability barrier facilitates the increased entrance of toxins and antigens, which in turn aggravates inflammation. In addition, ionizing radiation disturbs the antimicrobial properties of the epidermis and predisposes to infections.

In a cohort of patients receiving fractionated radiation therapy for breast cancer at doses ranging between 50–60 Gy, disruption of epidermal permeability barrier function was demonstrated [52]. In these studies, patients received tangential field irradiation to the chest wall by external beam, using photons (8MV) generated by a linear accelerator at single doses of 2 Gy, five times per week. Damage to the epidermis worsened over time, reaching a maximum after a mean of 27 days. In support of the concept that the barrier abnormality could drive inflammation, the onset of increased transepidermal water loss (TEWL), indicative of abnormal permeability barrier function, preceded the appearance of clinical symptoms, and maximal TEWL values preceded the peak of inflammatory skin changes. Moreover, an early increase in TEWL predicted a longer duration of skin symptoms. These studies identify increased TEWL as an early surrogate marker for radiation dermatitis and raise the possibility that preservation of permeability barrier function could decrease radiation-induced cutaneous damage [53]. It is likely that similar mechanisms apply to cutaneous damage observed following very low dose FGI procedures, but this has not formally been shown [54].

## Treatment of Radiation-Induced Skin Reactions

A considerable number of compounds have been tested for their ability to mitigate radiation dermatitis [55]. Previous publications demonstrated that topical treatment with corticosteroids improves epidermal barrier function and ameliorates the clinical severity of radiation injury to the skin [56, 57]. The benefits of topical corticosteroids are likely due to their anti-inflammatory effects. Inhibition of the radiation-induced cytokine secretion by glucocorticoids constitutes an important treatment principle for radiation-induced skin inflammation [56]. Yet, despite the short-term benefits, the adverse effect profile of glucocorticoids makes them less than optimal for therapy. Topical corticosteroids inhibit epidermal proliferation and differentiation by down-regulating lipid synthesis and also impair the permeability barrier function of the skin [58, 59].

Therefore, a considerable number of alternate emollients have been tested for their ability to mitigate radiation-induced skin injury [60]. However, the published data lack standardization across treatment protocols, which precludes an assessment of the comparative efficacy of these agents. Consequently, there currently is no entirely evidence-based gold standard for mitigating or treating radiation dermatitis, but topical corticosteroids in the inflammatory phase and emollients for longer term treatment are generally accepted. In the case of skin ulceration, treatment should follow the general principles of wound care, e.g., debridement and moist wound dressings (hydrogel, foam and hydrocolloid). In some cases, excision of the ulcer and skin grafting is necessary [45, 61].

## Dose Management Before, During and After the Procedure

The cornerstone of preventing radiation-induced skin injuries is minimizing the radiation dose and monitoring patients who are exposed to a cumulative skin dose above thresholds (Table 2) [4, 62–66]. This goal can only be achieved if the interventionalist is capable of identifying high-dose procedures and is attentive to individual risk factors in patients [62–65]. As mentioned before, a high body mass index (BMI) and previous radiation exposures are among the most important individual risk factors of patients. Thus, the interventionalist should not only focus on the patient’s discomfort and pathology, but should also thoroughly evaluate previous radiation exposures. Unless the skin dose from the planned procedure is very low or not affecting the previously irradiated skin area, the interventionalist has to consider an increased risk of skin injury.

During a complex interventional procedure, angiographic equipment can deliver more radiation to the skin than most radiation therapy units deliver in a single treatment session. Monitoring of radiation doses is, therefore, crucial [67]. Online dose monitoring is routinely performed in all patients undergoing FGI at the Department of Radiology in Innsbruck. During the last 2 years (2015–2016), we identified a *K*_ref_ > 3 Gy in 1,6% of all FGI and a *K*_ref_ > 5 Gy in 0,3%. The introduction of real-time dose monitoring decreased the number of high-dose procedures within the first year after introduction. The vast majority of high-dose procedures were neuroembolisations, pelvic and abdominal embolisations and endovascular abdominal aneurysm repairs (EVAR). A fluoroscopy time exceeding 60 min and a cumulative KERMA at refence point exceeding 5 Gy were quite common during complex endovascular aortic aneurysm repair requiring reconstruction of several aortic and/or iliac side branches. Thus, high-dose procedures are uncommon in routine practice, but do occur in complex endovascular procedures. The interventionalist has, therefore, to be aware of dose monitoring tools which nowadays are an integral part of modern angiographic equipment [5, 63, 65–68]. In state-of-the-art angiographic equipment, the interventionalist gets real-time information on dose in terms of the following parameters: KERMA at reference point (*K*_ref_), KERMA area product (KAP) and fluoroscopy time. In addition, the DICOM dose report, which becomes available at the end of the procedure, provides the number of runs, fluoroscopy time, the distribution of dose parameters between fluoroscopy and runs and the cumulative dose in terms of cumulative KERMA at reference point (*K*_ref_), cumulative KERMA area product (KAP) and cumulative fluoroscopy time. It is important to note that these displays are granted an uncertainty of ±35% [69]. *K*_ref_ and KAP are reasonably fit surrogate parameters for the estimation of the skin dose [70, 71]. If *K*_ref_ exceeds the thresholds level given in Table 1, patients should be counseled and followed as suggested by Balter et al. (Table 6) [21]. As evidenced by the Eurados WG-12 project K_ref_ correlates the best with skin dose in neuroembolisation and PCI, whereas in chemoembolisations KAP was the best skin dose indicator [71].

**Table 6:** General advice to be provided to patients and treating physicians

0–2 Gy	No need to inform patient, because there should be no visible effects
2–5 Gy	Advise patient that erythema may be observed but should fade with time
5–0 Gy	Advise patient to perform self-examination or ask a partner to examine for skin effects (erythema, itching) from about 2 to 10 weeks after the procedure
10–15 Gy	Medical follow-up is appropriate; skin effects may be prolonged, pain and necrosis may occur
>15 Gy	Medical follow-up is essential: radiation-induced wound may progress to ulceration and necrosis

Regarding dose management, the most efficient way to perform the procedure and to avoid excessive dose to a certain area of skin has to be considered. Thus, careful planning of the procedure and assigning an interventionalist who has sufficient experience and technical skills to handle the case is a first step in dose management [68]. Imaging during the intervention has to be optimized to match the appropriate image quality and the lowest possible dose. Table 7 gives an overview of important imaging parameters which influence patient dose.

**Table 7:** Important steps to minimize patient dose and to avoid radiation-induced skin injuries

Keep image receptor as close as possible to the patient
Maximize distance between patient and X-ray tube
Adapt tube settings (tube current, focal spot, filtration, exposure time and tube voltage) to patient size (usually done by automatic exposure control)
Use pulsed fluoroscopy, reduce frame rate and/or dose whenever possible
Use collimation, preferably virtual (off fluoroscopy)
Avoid direct magnification
Avoid angled views (remember that only 3 cm increase in body diameter doubles the skin dose)
Use road map or stored fluoroscopy loops instead of runs
Use last image hold instead of single shot
Avoid unnecessary cone beam CT, long fluoroscopy and multiple runs
Change beam entrance fields in long procedures if possible
Reduce to the minimum overlapping beam entrance fields in sequential FGI

Careful planning of the procedure, optimization of imaging parameters and training of staff are essential measures for the avoidance of an excessive dose to patients [8, 68, 72]. Routine evaluation of DICOM dose reports and real-time dosimetry are extremely helpful to optimize radiation protection of patients during interventional procedures. Some vendors even provide skin dose maps which can be of assistance in the identification of areas of skin at high risk [73].

In summary, modern angiographic equipment provides very helpful tools for decreasing and monitoring patient dose and, therefore, avoiding skin injuries.

The interventionalist performing potentially high-dose procedures shall inform patients about the risk of skin injuries. The report of the procedure should comprise dose metrics such as cumulative *K*_ref_ and cumulative KAP. If multiple procedures are performed on the same region of the body, a summary of all dose metrics shall be included in the final report. If a threshold level has been exceeded, the interventionalist should give a justification and document that the patient was informed about potential skin reactions and the necessity of the procedure. The interventionalist has to make sure that the patient is followed (Table 6) by a physician who is aware of the high radiation dose procedure and familiar with diagnosing radiation-induced skin injuries. Events of radiation doses above critical levels (Table 2) shall be discussed in a Quality Assurance–Peer Review committee including a qualified medical physicist. If possible and necessary, appropriate steps should be taken to avoid future events [74]. In most cases, an excessive patient skin dose can be avoided by simple and clinically feasible changes of practice.
